# Effect of metformin on serum interleukin-6 levels in polycystic ovary syndrome: a systematic review

**DOI:** 10.1186/1472-6874-14-93

**Published:** 2014-08-05

**Authors:** Xinghua Xu, Chigang Du, Qingmei Zheng, Lina Peng, Yuanyuan Sun

**Affiliations:** 1 Department of Gynecology and Obstetrics, Liaocheng People’s Hospital, 67 Dongchangxi Road, Liaocheng, Shandong Province 252000, China; 2 Department of Neurosurgery, Liaocheng People’s Hospital, Liaocheng, Shandong Province 252000, China; 3 Department of Gynecology, The Affiliated Hospital of Medical College, Qingdao University, Qingdao, Shandong Province 266500, China; 4 Department of Gynecology and Obstetrics, Dongchangfu District Maternal and Child Health Hospital, Liaocheng, Shandong Province 252000, China

**Keywords:** Polycystic ovary syndrome, Metformin, Interleukin-6, Chronic inflammation

## Abstract

**Background:**

Most women with polycystic ovary syndrome (PCOS) have insulin resistance, hyperinsulinemia, and elevated serum IL-6 levels. These elevated IL-6 levels may have links with insulin resistance and hyperandrogenism. Metformin may have beneficial effects on the chronic low-grade inflammatory background associated with PCOS.

**Methods:**

A systematic review was performed via PUBMED, EMBASE, and The Cochrane Library on PCOS studies published through November 30, 2013. Studies were selected that evaluated the effect of metformin on IL-6 levels in PCOS patients. Studies not containing adequate diagnosis information about PCOS or not excluding of other causes of hyperandrogenism were excluded.

**Results:**

Five studies met the inclusion criteria. Of these, one study reported a significant decrease in IL-6 levels after metformin treatment in women with PCOS. Two studies reported that treatment-related reductions in IL-6 levels were significantly correlated with insulin metabolism. In the remaining two studies, plasma IL-6 levels did not change following metformin treatment.

**Conclusions:**

Serum IL-6 levels of PCOS patients may be influenced by metformin. Early application of metformin therapy may relieve chronic low-grade inflammation in women with PCOS. However, further investigations with larger samples are needed to better understand the effects of metformin on IL-6 levels and chronic inflammation in PCOS.

## Background

Polycystic ovary syndrome (PCOS) is the most frequently encountered endocrinopathy and affects 5-8% of reproductive-age women [1]. It is characterized by chronic oligo-ovulation or anovulation, clinical or biochemical hyperandrogenism and polycystic ovaries [2,3]. This disorder is also associated with an increased risk of hyperinsulinemia, insulin resistance, type 2 diabetes mellitus, dyslipidemia, and cardiovascular diseases.

Interleukin-6 (IL-6), a pleiotropic cytokine, plays an important role in the endocrine system, especially as related to ovarian maturation and the processes of fertilization and implantation. IL-6 has also been shown to modulate ovarian development and function [4]. Therefore, IL-6 may be a key mediator of low-grade chronic inflammation in PCOS. Moreover, markers of chronic subclinical inflammation, such as IL-6, have been shown to be independent risk predictors for the development of type 2 diabetes [5]. Several studies have now documented an increase in IL-6 levels in PCOS patients [6,7]. Both Mohlig et al. and Gonzalez et al. have pointed out that elevated IL-6 levels may have links with insulin resistance and hyperandrogenism in PCOS [5,8]. Taken together, these studies support the hypothesis that PCOS increases the risk of diabetes by activating chronic inflammation [5].

More than half of women with PCOS have insulin resistance and hyperinsulinemia. These insulin abnormalities might play a significant role in the pathogenesis of PCOS, not only by influencing the reproductive abnormalities of PCOS, but also by amplifying metabolic defects [9,10]. Hyperinsulinemia may contribute to a hyperandrogenic state by increasing androgen production of theca cells and influencing hepatic production of sex hormone binding globulin, resulting in higher concentrations of free androgens [11]. Hyperinsulinemia is more common in affected women with reproductive morbidities such as gestational diabetes mellitus (GDM) and pre-eclampsia, which are also associated with insulin resistance [10]. Therefore, in PCOS patients, these findings have led to the development of an important therapeutic strategy based on insulin-sensitizing drugs, such as metformin.

Metformin has insulin-lowering effects by improving insulin sensitivity and, in turn, can decrease circulating androgen levels. In addition, it also plays a critical role in the treatment of PCOS because women with PCOS are at an increased risk of insulin resistance [12]. Indeed, metformin improves insulin-mediated glucose disposal in women with PCOS [10]. Thus, metformin has become one of the key drugs in the treatment of PCOS.

Considering the relationship between IL-6 levels and insulin resistance, metformin has the potential to affect serum IL-6 levels in PCOS patients. However, literature about this possible association is scarce and conflicting. In an attempt to elucidate the effect of metformin on IL-6 levels, we carried out this systematic review of the research literature.

## Methods

PUBMED, EMBASE, and The Cochrane Library were searched for articles published up to November 30, 2013, with no bars on foreign languages. The following key words were used: (PCOS OR polycystic ovary syndrome) AND (metformin) AND (interleukin-6 OR IL-6 OR inflammatory cytokine). References from the original articles found were also analyzed. Studies were included if they assessed the change of IL-6 levels before and after taking metformin in patients with PCOS.

Diagnostic criteria were considered valid if they conformed to 2003 Rotterdam [13], or 1990 National Institutes of Health (NIH) criteria [14]. Studies not containing adequate diagnosis information about PCOS or not excluding other causes of hyperandrogenism were ruled out. Case series or reports and reviews were also excluded.

## Results

**Figure 1:**
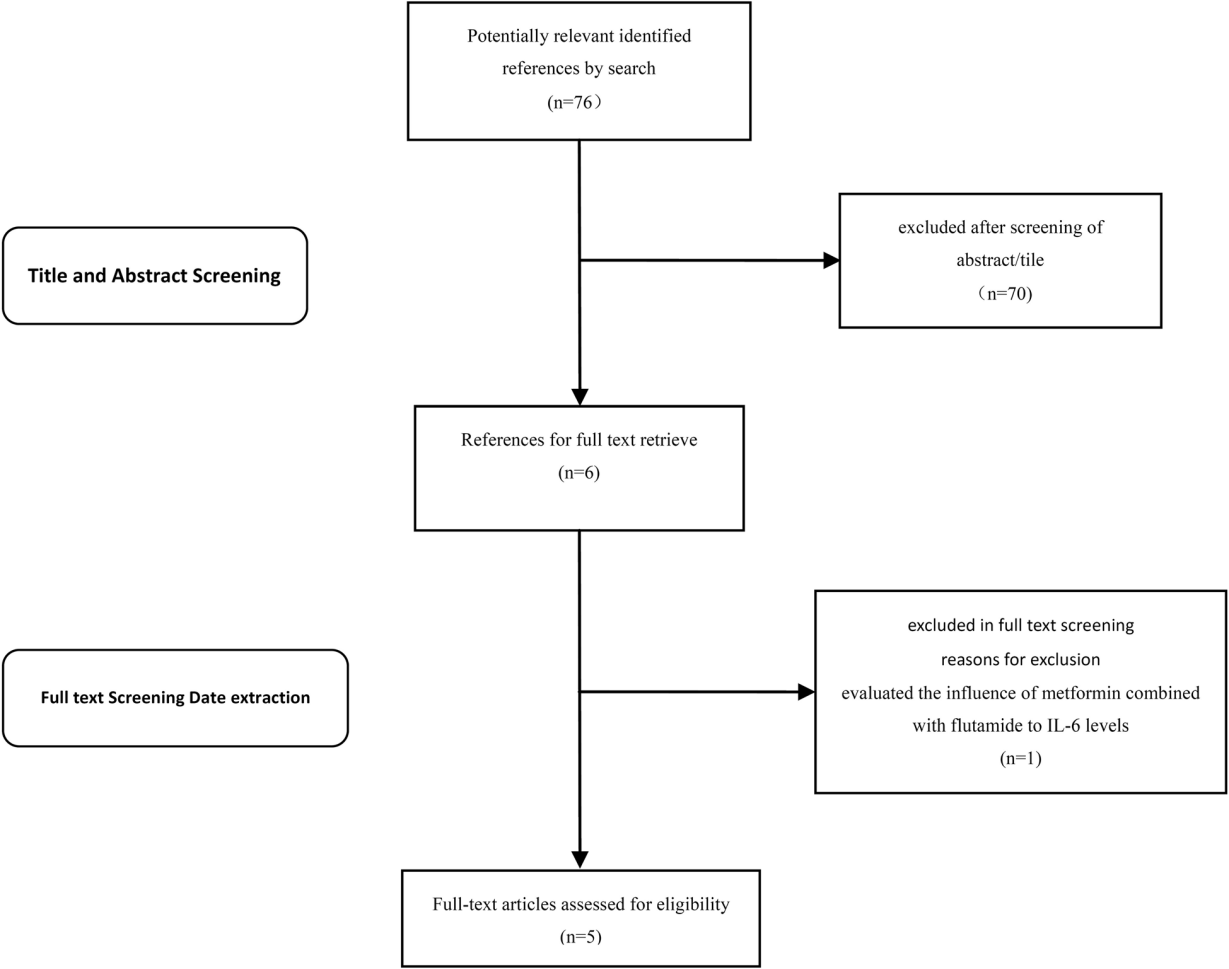
Flow chart of literature review.

In total, 76 articles were found. Seventy studies were ruled out based on their title or abstract. The six remaining studies evaluated the effect of metformin on IL-6 levels in PCOS patients. One study was excluded because it did not meet the objective of the present systematic review; this study by Ibáñez et al. [15] evaluated the influence of metformin combined with flutamide to IL-6 levels (Figure 1).

The five studies that were selected for further analysis were published between 2004 and 2013, all of them in English (Table 1). Three included European populations (Germany, Spain, Poland) [5,16,17] and one studied women of Asia (Taiwan) [6]. The last one study by Ciaraldi et al. was conducted in America [18]. Information on the ethnicity of participants was not provided for all the studies. Patients in these studies did not concurrently use insulin-sensitizing medication except metformin before or during the studies.

**Table 1:** Basic characteristics of PCOS patients and controls

**Author and year of publication**	**Diagnostic criteria for PCOS**	**population**	**Country**	**Age (years)**			**BMI (kg/m**^**2**^**)**^**)**^			**Abnormal glucose metabolism**	**Abnormal insulin metabolism**
				**Control**	**PCOS**	**P**	**Control**	**PCOS**	**P**		
Mohlig et al. 2004 [5]	clinical symptoms and on laboratory findings*	European	Germany	30.5 ± 1.5	28.9 ± 0.7	0.33	26.0 ± 1.3	30.3 ± 1.0	0.037	Fasting glucose	Fasting insulin
Jakubowska et al. 2008 [17]	2003 Rotterdam criteria	European	Poland	31.55 ± 7.36	28.24 ± 6.27	>0.05	32.94 ± 6.13	35.32 ± 5.07	>0.05	NON	Fasting insulin
Luque-Ramirez et al. 2010 [16]	hyperandrogenism, ovulatory dysfunction and exclusion of secondary etiologies	European	Spain	27 ± 5	24 ± 6	0.138	29 ± 8	30 ± 6	0.674	AUC glucose	NON
Lin et al. 2011 [6]	2003 Rotterdam	Asia	China, Taiwan	31.92 ± 0.58	27.67 ± 0.45	<0.0001	22.11 ± 0.45	24.25 ± 0.51	0.0025	PC sugar	2-hr insulin
Ciaraldi et al. 2013 [18]	1990 NIH	America	USA	32 ± 1	28 ± 1	<0.05	39.4 ± 2.2	35.6 ± 1.5	>0.05	2 h glucose	NON

*AUC*: area under the curve of the oral glucose tolerance test; *NON*: no difference was reported.

*Clinical symptoms and on laboratory findings: clinical symptoms, i.e. oligo- or amenorrhea and hirsutism, and on laboratory findings, i.e. serum androgen levels above the upper limit of normal for the respective assay.

Sample sizes were varied across studies. Across all of the five studies, the total number of PCOS women treated with metformin was 191, and there were 183 control patients. Rotterdam diagnostic criteria were used in two studies [6,17]; while NIH criteria were used in one article [18]. The authors of the other two studies primarily relied on their own criteria; however, these patients also met the NIH criteria. Basic characteristics of PCOS patients and controls are presented in Table 1.

Lin and colleagues observed a significant decrease in IL-6 levels after metformin treatment in PCOS women (IL-6 level decreased from 28.05 ± 3.26 to 22.04 ± 2.76 pg⁄mL after metformin treatment in PCOS women, p = 0.0342) [6]. Both Luque-Ramirez et al. [16] and Ciaraldi et al. [18] suggested that treatment-related reductions in IL-6 levels were significantly correlated with insulin metabolism. Luque-Ramirez et al. [16] reported that the decrease in serum IL-6 levels in patients treated with metformin was inversely correlated with the increase in the insulin sensitivity index (r = − 0.579, p = 0.048; intention-to-treat analysis, r = − 0.687, p = 0.001) and positively correlated with the decrease in the insulin secretion index (r = 0.577, p = 0.050; intention-to-treat analysis, r = 0.679, p = 0.001). Ciaraldi et al. [18] also reported that treatment-related reductions in IL-6 levels were significantly correlated with drops in fasting insulin (data was not shown in their article) (Table 2).

**Table 2:** Characteristics of studies evaluating effect of metformin on IL-6 levels of polycystic ovary syndrome

**Author and year of publication**	**No. of cases**	**No. of controls**	**Dosage of metformin**	**IL-6 level before treatment (pg/ml)**	**IL-6 level after treatment (pg/ml)**	**P**	**Outcomes**
Mohlig et al. 2004 [5]	9 obese, insulin-resistant PCOS patients	20 health women	850 mg, three times daily, 6 months	1.72 ± 0.30	1.91 ± 0.31	0.515	After 6 months therapy, IL-6 concentrations remained largely unchanged
Jakubowska et al. 2008 [17]	29 obese PCOS patients	29 healthy, premenopausal volunteers matched for BMI.	500 mg, twice daily, 6 months	34.32 ± 8.28	33.36 ± 8.28	>0.05	plasma IL-6 levels did not change during therapy
Luque-Ramirez et al. 2010 [16]	19 PCOS women	18 health women	850 mg, twice daily, 24 weeks	1.0 ± 0.9	0.7 ± 0.5	0.56	The decrease observed in serum IL-6 levels in the patients treated with metformin correlated inversely with the increase in the insulin sensitivity index and directly with the decrease in the insulin secretion index
Lin et al. 2011 [6]	129 PCOS women	109 control women	500 mg, three times daily, 12 weeks	28.05 ± 3.26	22.04 ± 2.76	0.0342	There was a significant decrease in IL-6 level after metformin treatment in PCOS women.
Ciaraldi et al. 2013 [18]	5 PCOS women	7 control women	2 g/d, 6 months	Data was not showed	Data was not showed	Data was not showed	Treatment-related reductions in IL-6 levels were significantly correlated with falls in fasting insulin

Results for the other two studies were inconclusive. Mohlig et al. [5] reported that IL-6 concentrations did not change after metformin treatment (34.32 ± 8.28 pg/ml vs 33.36 ± 8.28 pg/ml, p > 0.05). Jakubowska et al. [17] was to the only study to focus on a sample of obese women with PCOS. While there was a significant decrease in BMI (35.32 ± 5.07 kg/m^2^ vs. 33.49 ± 4.80 kg/m^2^, p = 0.00002) and waist circumference (106.84 ± 12.69 cm vs. 101.80 ± 11.75 cm, p = 0.005) before and after metformin treatment, the change in pre-to-post IL-6 concentrations was not significant (1.72 ± 0.30 pg/ml vs. 1.91 ± 0.31 pg/ml, p = 0.515) (Table 2).

Across all five studies, there was substantial variation in drug dosage, route of administration of metformin, patient BMI, and patient levels of insulin metabolism before metformin treatment. As such, a true meta-analysis was not performed across all of these variables because such analysis would not have yielded clinically meaningful results. Thus, the body of evidence was not consistent or strong and ore empirical research is needed in this area.

## Discussion

As a complex and heterogeneous disorder, the pathogenesis of PCOS has remained largely unknown until now. Over the past few decades, PCOS researchers have focused their attention on the state of chronic low-grade subclinical inflammation and how it relates to the endocrine, metabolic, and reproductive disturbances in PCOS patients. Genetic variants in the genes encoding inflammation-related mediators underlie the development of PCOS and their interaction with environmental factors may contribute to the heterogeneous clinical phenotypes of PCOS [19]. Insulin resistance has been increasingly recognized as having an important role in inflammatory pathways [9], and metformin may play an important role in improving low-grade inflammation in PCOS. Hence, the present study systematically reviewed the empirical research literature relating metformin to serum IL-6 levels in PCOS.

With regard to young and lean PCOS patients, elevated IL-6 levels have been observed to decrease after metformin treatment. Lin et al. demonstrated that IL-6 may be an early low-grade chronic inflammatory marker in PCOS [6], a finding that is consistent with other evidence that IL-6 may be a key mediator of low-grade chronic inflammation in PCOS [4]. This research on IL-6 is shedding further light on the pathogenesis of PCOS and the long-term cardiovascular disease risk associated with PCOS [20]. Therefore, strategies ameliorating inflammation may be useful for the management of PCOS and associated conditions [19].

Modern chronic drug treatment for PCOS patients is typically based on the administration of oral contraceptives, antiandrogens, and/or insulin sensitizers [16]. As a biguanide that improves insulin sensitivity, metformin has been extensively evaluated in PCOS [11] and has been shown to play a critical role in improving low-grade inflammation in PCOS [9]. Lin et al. found there was a significant decrease in IL-6 levels after metformin treatment in PCOS women [6]. Luque-Ramirez et al. confirmed that metformin may have beneficial effects on the inflammatory background associated with PCOS [16]. Ciaraldi et al. indicated that treatment-related reductions in IL-6 levels were significantly correlated with drops in fasting insulin levels [18]. However, Mohlig et al. and Jakubowska et al. failed to find any changes of plasma IL-6 levels with metformin therapy in PCOS patients [5,17]. Thus, considering the fact that PCOS is a very heterogeneous disease, it is difficult to study the exact influence of metformin on the inflammation state of PCOS women. In order to reduce the influence of heterogeneity, studies between different subgroups are required. Nevertheless, the number of relevant studies reviewed here are limited and they did not carry out analyses of different PCOS subgroups. Ignoring the heterogeneity of PCOS is perhaps one of the reason why discrepant results have been reported in the literature.

Obesity is frequently present in women with PCOS. Continuous release of inflammatory mediators such as IL-6 perpetuates the inflammatory condition associated with obesity in PCOS, possibly contributing to insulin resistance and other long-term cardiometabolic risk factors [19]. Linear regression models have revealed that BMI is the dominant parameter determining IL-6 and C-reactive protein (CRP) values in PCOS [5]. Gözdemir et al. suggested that obesity was the principle mechanism of chronic inflammation and insulin resistance in PCOS patients. CRP and IL-6 should be used to predict and follow the risk of CVD development in PCOS cases [21]. However, six months of metformin therapy with obese PCOS women in the study of Jakubowska et al. were insufficient to reliably decrease their IL-6 levels [17]. BMI and fat mass may improve while elevated IL-6 levels are maintained [5]. Among the other studies included in this systematic review, calculations for determining the relationship between BMI and IL-6 were not explicitly made [6,16,18], so further investigations are needed to address these interrelationships.

Another possible reason for the mixed results across the literature regarding the influence of metformin on the level of IL-6 could be ethnic and geographic variation. Discrepancies could also be explained by the small sample size of some included studies, which may result in substantial errors from estimation. In view of the current data, most sample sizes are small [5,16-18]. Only the study of Lin et al. involved a large sample with 129 PCOS women and 109 controls [6]. Population stratification and sample size are important issues to be concerned in human surveys.

Despite on the above considerations, we endorse that metformin does seem to significantly influence IL-6 levels. PCOS women with early application of metformin therapy may improve their state of chronic inflammation. However, the literature shows conflicting results because of different study designs. Future studies with diverse populations are needed with larger samples to better understand the effect of metformin on IL-6 levels and chronic inflammation of PCOS women.

## Conclusions

In the present study, the relationship between the IL-6 levels and metformin in PCOS was systematically reviewed. Across several studies, the data support that metformin may influence IL-6 levels and ameliorate the state of chronic inflammation in PCOS women that receive early metformin therapy. Considering the conflicting results reported in the literature, however, further investigations are necessary with larger samples to understand better the effect of metformin on the IL-6 levels and chronic inflammation in PCOS.

## Abbreviations

PCOS: Polycystic ovary syndrome; IL-6: Interleukin-6; BMI: Body mass index; GDM: Gestational diabetes mellitus; NIH: National Institutes of Health; CVD: Cardiovascular disease.

## Competing interests

The authors declare that they have no competing interests.

## Authors’ contributions

All the authors contributed to the conception of the review. XX and DC performed literature search, selected the abstracts and abstracted the data. ZQ resolved the discrepancies between the two reviewers (XX and DC) in the selection of the study of interest. PL resolved the dicrepancies between the two reviewers (XX and DC) in the abstraction of the data from the study of interest. XX prepared the first draft of the manuscript and performed subsequent amendments. ZQ and SY reviewed the manuscript. All authors read and approved the final manuscript.

## Pre-publication history

The pre-publication history for this paper can be accessed here:

http://www.biomedcentral.com/1472-6874/14/93/prepub
